# Sunlight exposure practice and its associated factors among infants in Ethiopia, systematic review and meta-analysis

**DOI:** 10.1371/journal.pone.0300598

**Published:** 2024-11-04

**Authors:** Fanos Yeshanew Ayele, Natnael Kebede, Asnakew Molla Mekonen, Mengistu Mera Mihiretu, Yawkal Tsega, Elsabeth Addisu, Niguss Cherie, Tesfaye Birhane, Chala Daba, Ahmed Hussien Asfaw, Zinet Abegaz, Abel Endawekie, Anissa Mohammed, Dagnachew Melak, Fekade Demeke Bayou, Husniya Yasin, Aregash Abebayehu Zerga, Fikre Bayu Gebeyehu, Birhanu Wagaye

**Affiliations:** 1 Department of Public Health Nutrition, College of Medicine and Health Sciences, Wollo University, Dessie City, Ethiopia; 2 Department of Health Promotion, School of Public Health, College of Medicine and Health Sciences, Wollo University, Dessie City, Ethiopia; 3 Department of Health System and Management, School of Public Health, College of Medicine and Health Sciences, Wollo University, Dessie City, Ethiopia; 4 Department of Reproductive and Family Health, School of Public Health, College of Medicine and Health Sciences, Wollo University, Dessie City, Ethiopia; 5 Department of Environmental Health, College of Medicine and Health Sciences, Wollo University, Dessie, Ethiopia; 6 Department of Epidemiology and Biostatistics, School of Public Health, College of Medicine and Health Sciences, Wollo University, Dessie, Ethiopia; 7 Department of Anatomy, School of Medicine, College of Health Sciences, Addis Ababa University, Addis Ababa, Ethiopia; Haramaya University Faculty of Health Sciences: Haramaya University College of Health and Medical Sciences, ETHIOPIA

## Abstract

**Background:**

Lack of sunlight exposure is the primary reason for the worldwide epidemic of vitamin D deficiency. There was a meta-analysis conducted under the title of Knowledge and Practice of Mothers towards sunshine exposure of their children in Ethiopia. However, studies conducted on factors associated with sunlight exposure practice among infants showed non-conclusive and inconsistent findings. Hence, this systematic review and meta-analysis were conducted to estimate the pooled prevalence of good sunlight exposure practice and its associated factors among infants in Ethiopia.

**Methods:**

All articles were systematically searched by PubMed, Hinari, Global Health and CINAHL, Epistemonikos, African Journal of Online (AJOL), Google Scholar and Google. All studies, until the end of May 30, 2023, were included in this review. Pure qualitative studies and studies in which the outcome was not reported were excluded from the review. The Preferred Reporting Items for Systematic Reviews and Meta-Analyses guideline was used. Meta-analysis was conducted by using STATA 17 software. Forest plots were used to present the pooled prevalence of good sunlight exposure practices. A random effect model was used to compute the pooled prevalence, while subgroup analysis was performed to identify the possible source of heterogeneity. Publication bias was assessed by begg’s and Egger’s tests. We use Trim and fill analysis was used to treat the publication bias.

**Results:**

This review involved 14 studies and 6,121 participants. The pooled prevalence of good sunlight exposure practice among infants in Ethiopia was 53.46% (95%CI: 45.98, 60.95). Mothers had PNC follow-up [OR = 2.22 (95% CI: 1.31, 3.47)], mothers with secondary and above educational status [OR = 4.17, (95% CI: 1.73, 10.06)], employed mothers [OR = 3.72, (95% CI: 2.71, 5.11)], urban residence [OR = 2.67, (95% CI: 1.17, 6.08)] and not fear of sunlight exposure [OR = 4.08, (95% CI: 1.44, 16.00)] were positively associated with good sunlight exposure practice.

**Conclusions:**

The pooled prevalence of good sunlight exposure practices among infants in Ethiopia is low. Had postnatal care follow-up, being urban residents, mother’s employment status, mother’s educational status and not fear of sunlight exposure were independent factors of good sunlight exposure practice among infants. Therefore, health professionals create awareness for mothers to increase postnatal follow-up, and the importance of health education especially for rural residents.

## Background

In 1919, the first scientifically documented the health importance of sunlight exposure to prevent and cure rickets. Vitamin D can be synthesized in the skin by a photosynthetic reaction triggered by exposure to sunlight [1]. When the skin is stimulated with ultra-violate A (UVA) radiation, nitric oxide is released which is used to stimulate vasodilation and decrease blood pressure [2]. Additionally, human skin produces beta-endorphin in response to ultra-violate B (UVB) exposure [3]. These opioid peptides have the result of increasing a feeling of well-being, boosting the immune system, relieving pain, promoting relaxation, wound healing, and cellular differentiation [4].

The amount of sunlight exposure needed to synthesize adequate vitamin D depends upon the type of skin, [5] the time of the day, the month of the year, and the latitude [6]. The duration needed in an individual with dark skin is about ten times that in fair-skinned individuals. It has been suggested that exposure to sunlight for 5 minutes to 5 hours per day (depending upon the above-mentioned factors) may be sufficient to synthesize our daily requirement of vitamin D [6, 7].

According to studies conducted in Saudi Arabia, Sakayara, and Brazil, mothers’ poor practice of sunlight exposure to their children ranges from 66.7% to 75% [8–10]. According to a study conducted in Egypt, 27.3% of mothers had poor practices when exposing their infants (21). In Ethiopia, mothers’ poor practice of sunlight exposure to their babies ranges from 34.3% to 67.4 [5, 11–14].

Globally, lack of sun exposure has been identified as the main cause of rickets [15]. Recently, vitamin D deficiency has been reported to be a public health problem worldwide, regardless of abundant sunlight in many countries. According to the World Health Organization, approximately one billion people are vitamin D deficient, and the deficiency is most prevalent in infants regardless of topography, climatic condition, ethnicity, and age (13,25). Rickety is common in Sub-Saharan African countries, including Ethiopia, and its prevalence ranges from 4% to 41% [16–19].

Health education was adopted as the main strategy to combat rickets in Ethiopia to change maternal behaviour to appropriately expose their infants to sunlight. However, the implementation of the strategy has remained inadequate, and health messages lack focus on factors that influence maternal practice excluding infants from getting adequate sunlight. This was largely because of a lack of adequate information on the determinants of this particular risk behaviour among Ethiopian mothers [20].

Determining the pooled magnitude and associated factors of sunlight exposure practice among infants is vital to designing effective interventions to reduce inadequate sunlight exposure practice. There was no single conclusive finding regarding factors affecting sunlight exposure practice in Ethiopia. Even though the pooled prevalence of sunlight exposure practices in Ethiopia, this study includes only published studies and studies with the title of knowledge and practice of sunlight exposure. In addition, public health experts and policymakers who are working with infants need updated evidence regarding sunlight exposure practices. Therefore, this review aimed to estimate the pooled prevalence of good sunlight exposure practice and its associated factors among mother-infant pairs in Ethiopia.

## Materials and methods

### Registration

The preferred reporting items for systematic review and meta-analysis (PRISMA) guidelines were used to perform this systematic review and meta-analysis [21]. It has been registered in the International Prospective Registry of Systematic Reviews (PROSPERO) with a specific registration number of CRD42022382520.

### Search strategy and studies identification

The studies were retrieved compressively from both published and unpublished articles. The published articles were searched by using different databases such as All articles were systematically searched by PubMed, Hinari, Global Health and CINAHL, Epistemonikos, African Journal of Online (AJOL) and unpublished literature sources like Google Scholar and Google. All relevant articles were searched and retrieved by using a combination of search terms/ keywords like; “Sunlight exposure practice”, “sunshine exposure practice”, “sunlight exposure”, “determinant”, “Predictors”, “factors”, “associated factors”, “infants, “infant-mother pair”, “Ethiopia’‘ using Boolean operators "OR" or "AND" as appropriate and the search was done by two authors independently FY, NK, YT, FD, EA, and AA).

### Inclusion and exclusion criteria

All studies, irrespective of data collection and publication year until the end of May 30, 2023, were included in this review. This review included published studies with only the English language and unpublished studies that were conducted on the prevalence and factors associated with sunlight exposure practice among infants in Ethiopia. All observational studies measured sunlight exposure practice and associated factors among infants were included in this review. However, pure qualitative studies and quantitative studies not report outcomes were excluded from the review.

### Outcome variable

The primary outcome of this review was the prevalence of good sunlight exposure practices among infants. The second objective was the associated factors of good sunlight exposure practices conducted in Ethiopia. The odds ratio/ 2x2 contingency was extracted for associated factors of good sunlight exposure practice.

### Study selection, risk of bias assessment, and data extraction

Those studies searched from selected databases were transferred to End Note, and duplicate files were excluded. The rest articles and abstracts were separately screened by two groups (FY, YT, NK, FD, AA, HY, AM, AH, AE, FB and EA) for inclusion in the full-text appraisal. The differences between reviewers were managed by discussion, and disagreement was handled by the third party (CB, NC, TB, ZA, MM, AMM, DM, FB and BW). The quality of articles was evaluated using the Joanna Briggs Institute (JBI) critical appraisal checklist [22]. Two reviewers have separately assessed articles before inclusion for review.

Nineteen authors (FY, YT, NK, FD, AA, HY, AM, AH, AE, EA, CB, NC, TB, ZA, MM, AMM, DM, FB and BW) independently retrieved all the necessary data using Microsoft Excel 2010 sheet. The data extraction tool contains information on the Author’s name, year of publication, study area, response rate, sample size, study quality score, and prevalence.

### Statistical methods and analysis

The statistical analysis was conducted using STATA 17 software. A Forest plot was used to display the magnitude of sunlight exposure practice among infants in Ethiopia. Due to the significant presence of heterogeneity among studies, the random effect model of analysis was applied. The pooled prevalence of sunlight exposure practice among adolescents was presented with a 95% CI. The heterogeneity test of involved studies was measured by using the inverse variance (I^2^) statistics. The value of I ^2^ was interpreted as 25% as low, 50% as medium, and 75% as high heterogeneity [23]. Subgroup and sensitivity analysis was also conducted by different study characteristics such as sub-region of Ethiopia (Amara or other), sample size (small or large), and residence (urban or rural). The presence of publication bias was assessed using Begg’s test and the Egger regression asymmetry test [24]. When the p-value was less than 0.05, it declared the presence of a publication bias.

## Results

### Study selection

**Fig 1:**
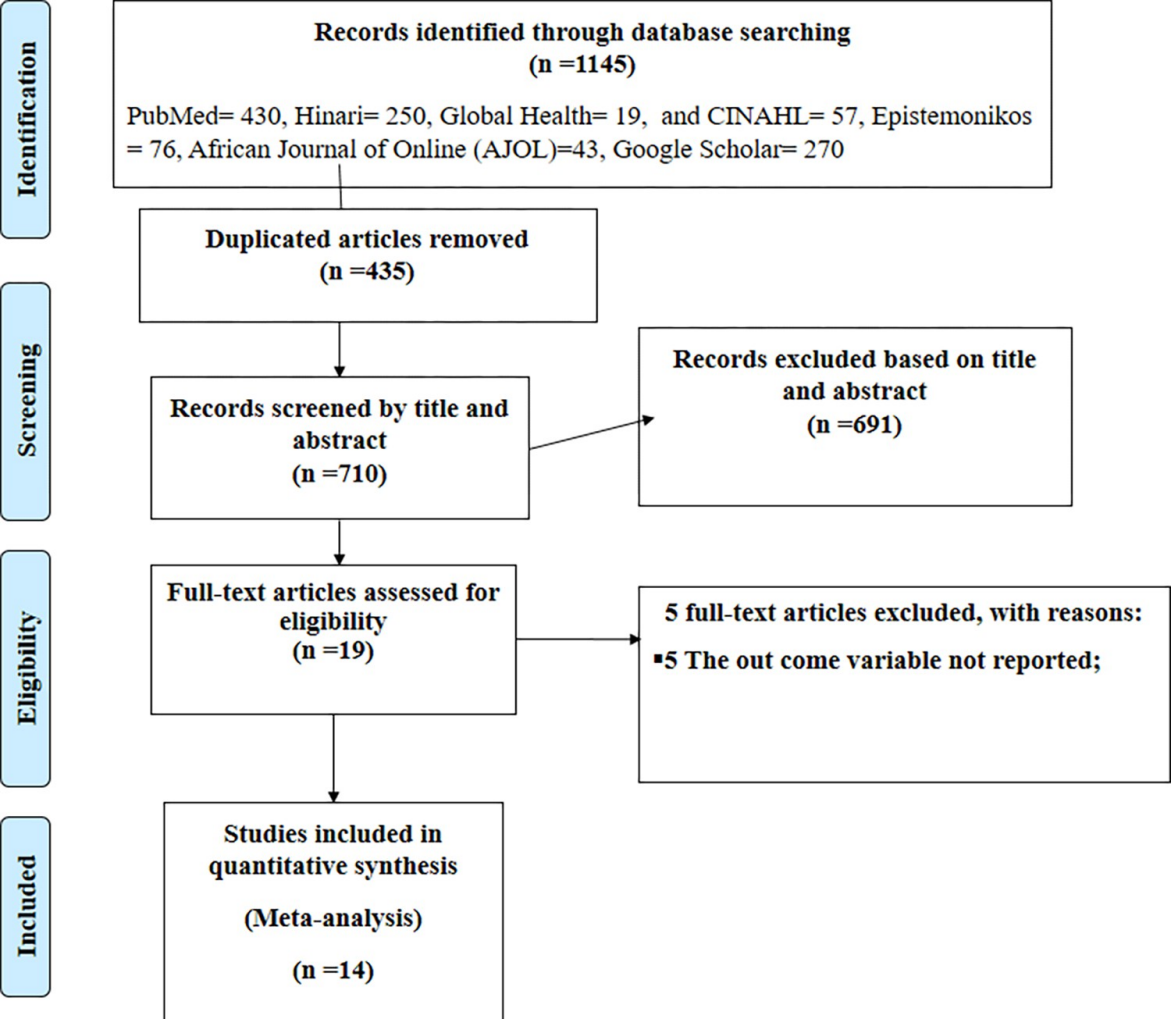
PRISMA flow diagram of the included studies for meta-analysis of dietary diversity practice and associated factors among adolescents in Ethiopia.

This systematic review and meta-analysis identify 1145 published and unpublished studies conducted on sunlight exposure practice and associated factors among infant-coupled mothers in Ethiopia. Then 435 were excluded due to duplication, and the remaining 691 studies were excluded due to their titles and abstracts. Nineteen full-text articles were assessed for eligibility. From these, five full-text articles were removed for prior criteria, and a total of 14 studies were included in the review (Fig 1).

### Characteristics of included studies

A total of 14 studies [5, 11, 13, 14, 25–34] with 6,121 study participants were included to estimate the pooled prevalence of sunlight exposure practice and associated factors among infant mothers pair in Ethiopia. The sample size of the studies ranged from 105 study participants in a study conducted in the Debre Tabor [14] to 866 participants in a study conducted in Dejen Woreda [29]. In this meta-analysis, four regions from nine regions of the country were represented; two studies from Addis Ababa [25, 26], eight studies from Amhara [5, 11, 14, 28–32], four studies from SNNP [13, 27, 34, 35] one from Oromo [33]. The studies were conducted from 2015 to 2021 in different regions of the country (Table 1).

**Table 1:** Summary characteristics of studies included in the meta-analysis of the prevalence of dietary diversity practice among adolescents in Ethiopia.

Authors and Publication year	Study year	Study area	Study design	Sample size	Prevalence (%)		Quality score
					Good sunlight exposure practice	poor sunlight exposure	
Dessalegn Et al, 2020	2020	Addis Ababa	Institutional based cross-sectional	346	54.6	45.4	80%
Bedaso et a, 2019	2018	SNNPR	Institutional based cross-sectional	313	46.33	53.67	85%
Teklehaimanot et al, 2021	2019	Debre Birhan	Community-based cross-sectional	530	65.66	34.34	78%
Bekalu et al, 2022	2017	Dejen Woreda	Community Based cross sectional	866	44	56	68%
Goshiye et al., 2023	2021	Dessie town	Institutional based cross-sectional	455	38.7	61.3	71%
Abebe Abate et al, 2016	2015	Dembia district	Institutional based cross-sectional	345	43.19	56.81	82%
Ashebir YG et al., 2022	2020	Addis Ababa	Institutional based cross-sectional	420	27.14	72.86	75%
Feleke DG, et al, 2020	2018	Debre Tabor	Institutional based cross-sectional	105	59.05	40.95	60%
Gedamu H, et al, 2019	2018	Farta	Institutional based cross-sectional	339	45.72	54.28	72%
Andargie W,et al, 2021	2021	Bahir Dar city	Institutional based cross-sectional	414	75.12	24.88	80%
Mengistu TG et al,2022	2020	Wolkite	Institutional based cross-sectional	220	35.75	64.25	75%
Hora A, 2022	**2022**	Adami Tulu Jido-Kombolcha	community-based cross-sectional	575	54.43	45.57	85%
Kindus S, 2018	2018	South Achefer	community-based cross-sectional	377	70.56	29.44	71%
Bezabih AS et al(2021)	2019	Yirgalem General Hospital.	Institutional based cross-sectional	277	54.5	45.5	73%
Tadesse A et al (2023)	2019	Mettu district	community-based cross-sectional	346	57.7	254	86%

### Prevalence of good sunlight exposure practice

**Fig 2:**
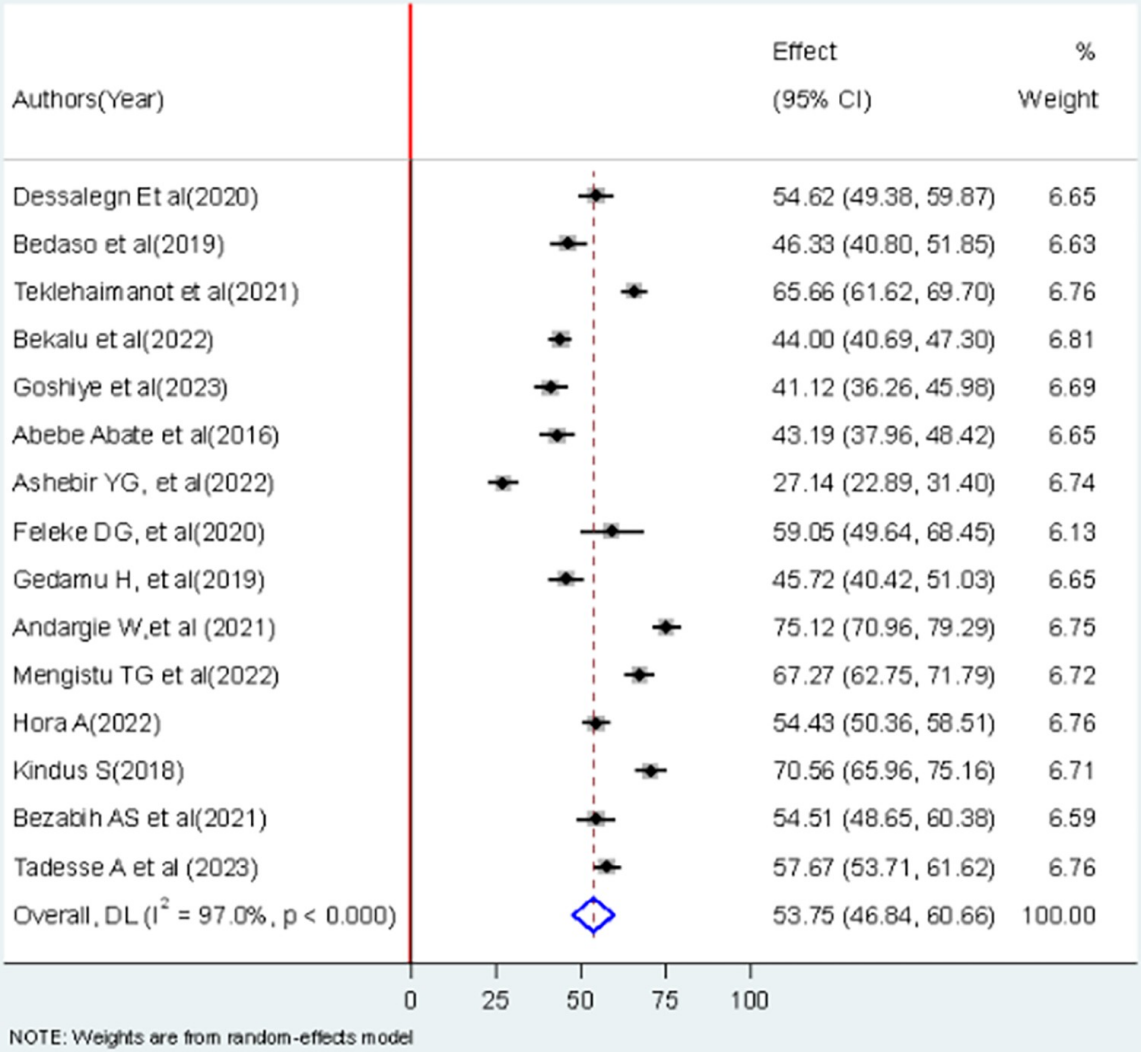
Forest plot of the prevalence of good sunlight exposure practice among mother infant pairs in Ethiopia.

**Fig 3:**
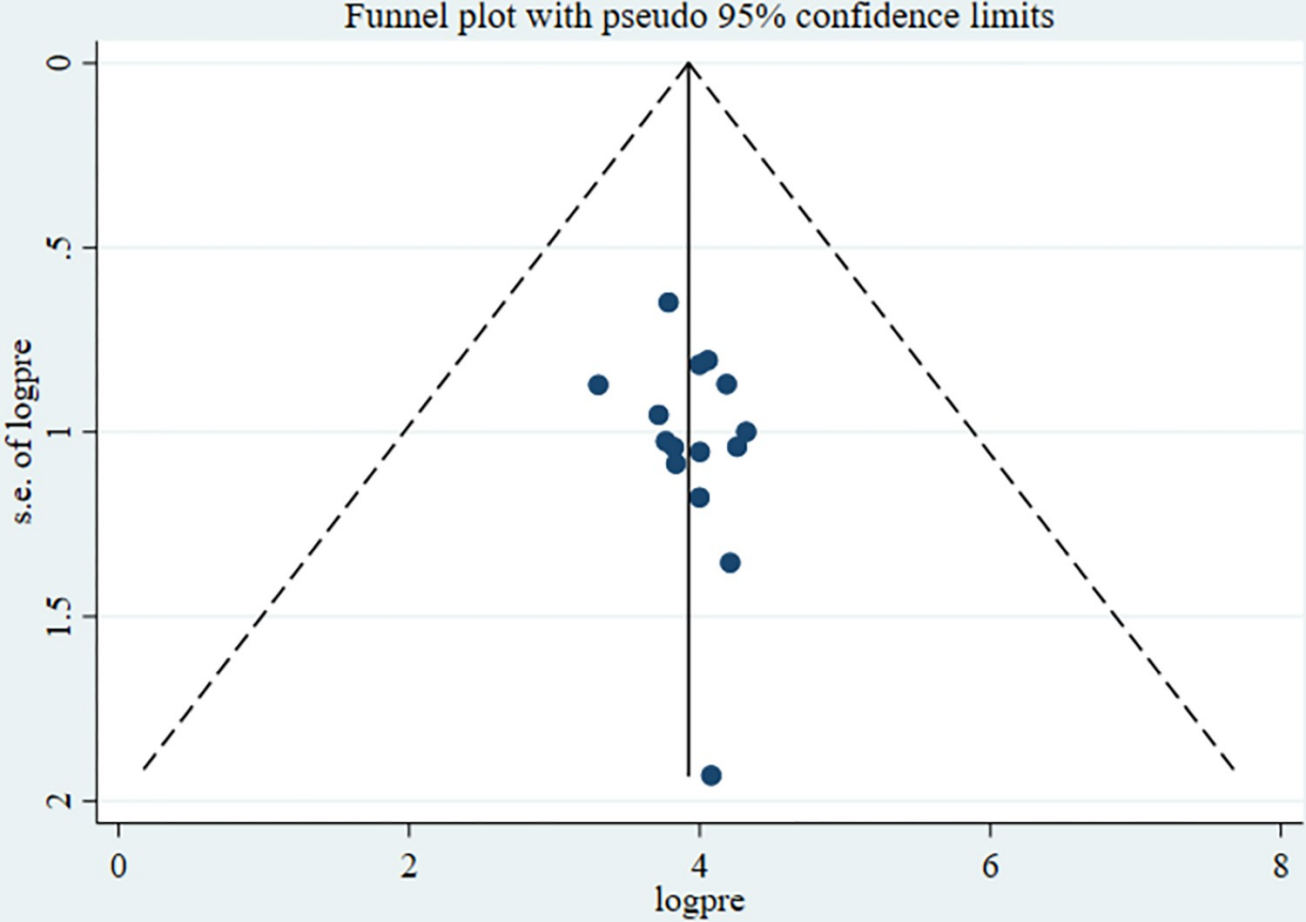
Funnel plot for the prevalence of good sunlight exposure practice among mother-infant pairs in Ethiopia.

The overall pooled lifetime prevalence of good sunlight exposure practice among infants in Ethiopia was 53.75% (95%CI: 46.84, 60.66). The highest lifetime prevalence of good sunlight exposure practice was reported in a study done in Bahir Dar City. The study showed that 75.12% of infants had experienced good sunlight exposure practice [28]. The lowest prevalence of good sunlight exposure practice was 27.14% among infants in Addis Ababa [26]. Significant heterogeneity was observed among included studies in the meta-analysis, I ^2^ = 97.2%, p < 0.001 (Fig 2). The funnel plot showed a symmetrical appearance (Fig 3).

**Fig 4:**
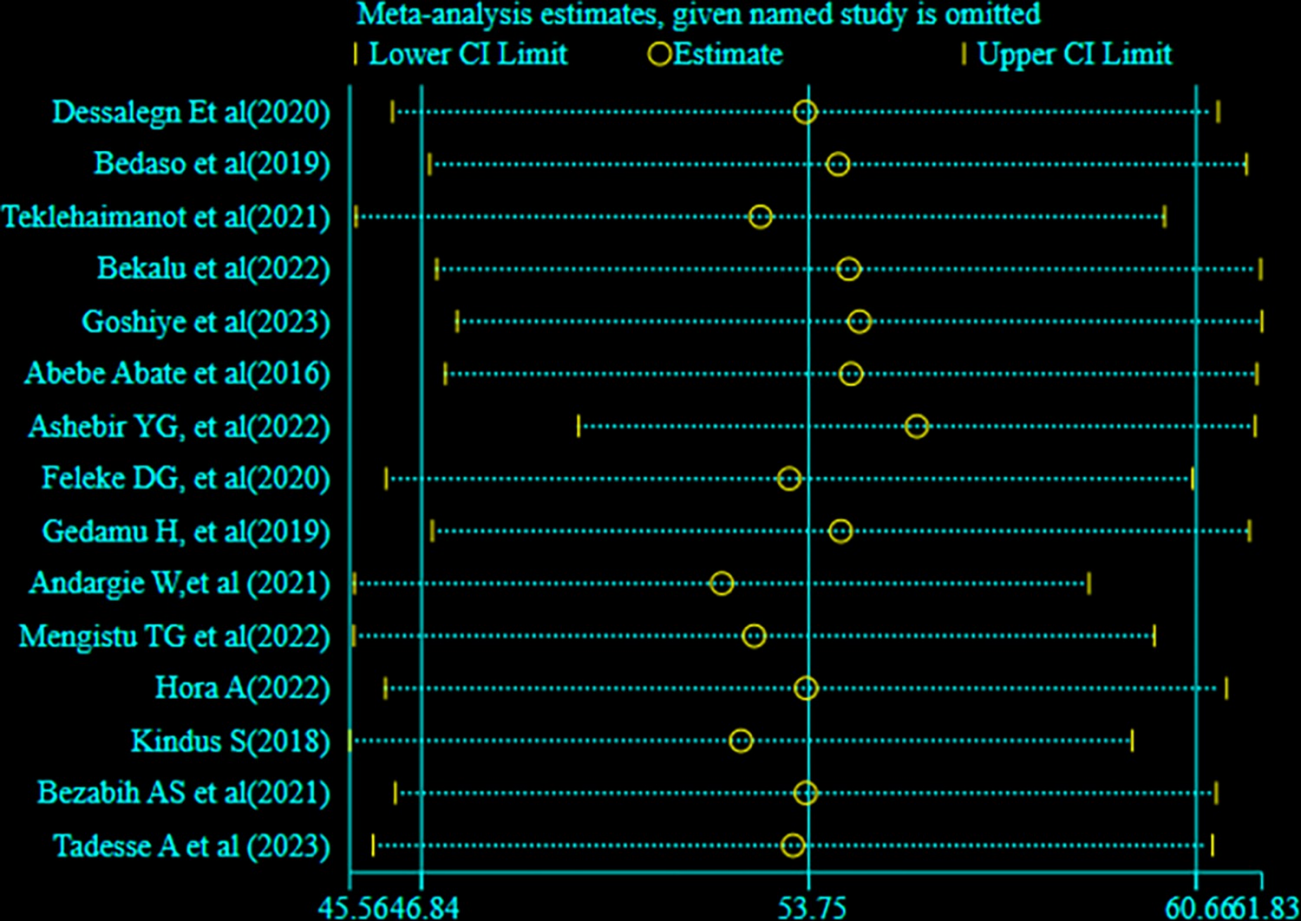
Sensitivity analysis of the prevalence of good sunlight exposure practice among mother-infant pairs in Ethiopia.

The Egger’s regression asymmetry test also showed non-significant publication bias, p-value **= 0.287.** Sensitivity analysis was conducted to identify the possible source’s source of bias, but no outlier study potentially shifted the primary pooled estimates (Fig 4).

### Subgroup analysis

The subgroup analysis was conducted by region, sample size, and study design to identify the discrepancy across the studies. Even though heterogeneity still existed in the subgroup analysis of all the above-mentioned factors, the prevalence of good practice of sunlight exposure was significantly higher among studies conducted in the SNNP region [56.57, 95% CI: (48.28., 64.86)] as compared to studies conducted in another region. Also, the prevalence of good sunlight exposure practice was a significant difference between community-based study design [58.60, 95% CI: (46.52, 70.68)] and institutional study design [51.37 95%CI: (41.44, 61.30)] (Table 2).

**Table 2:** Subgroup analysis of the dietary diversity practice among adolescents in Ethiopia.

Sub-groups	Number of studies	Total sample	Prevalence (95% CI)	Heterogeneity	
				I^2^	p-value
By region					
Amara	8	3,370	55.55 (45.40, 65.69)	97.4	< 0.001
Other	5	2,151	51.71 (41.79, 61.62)	96.8	<0.001
By Region					
Amhara	8	3,370	55.55 (45.40, 65.69)	97.4	< 0.001
SNNP	4	1410	56.57(48.28, 64.86)	94.2	< 0.001
Addis Ababa	2	766	40.84(13.91, 67.77)	99.4	< 0.001
Oromo	1	575	54.43(50.36, 58.51)	0.0%	< 0.001
By sample size					
Small	9	2,716	53.57(45.99, 61.15)	94.5%	< 0.001
Large	5	2, 805	54.00 (41.10, 66.90)	98.7%	< 0.001
By Study design					
Institutional based	9	3,173	51.37 (41.44, 61.30)	97.3	<0.001
Community-based	5	2948	58.40 (49.05, 67.75)	97.3	< 0.001
Total	7	6,121	53.40 (49.05, 67.75)	97.0	< 0.001

### Associated factors of sunlight exposure practice among infants

In this study, we assessed factors associated with good sunlight exposure practice. A separate analysis was conducted for each factor which was considered in this meta-analysis. Variables assessed with the sunlight exposure practice were: PNC follow-up, maternal educational status, maternal occupational status, residence, and fear of sunlight exposure.

**Fig 5:**
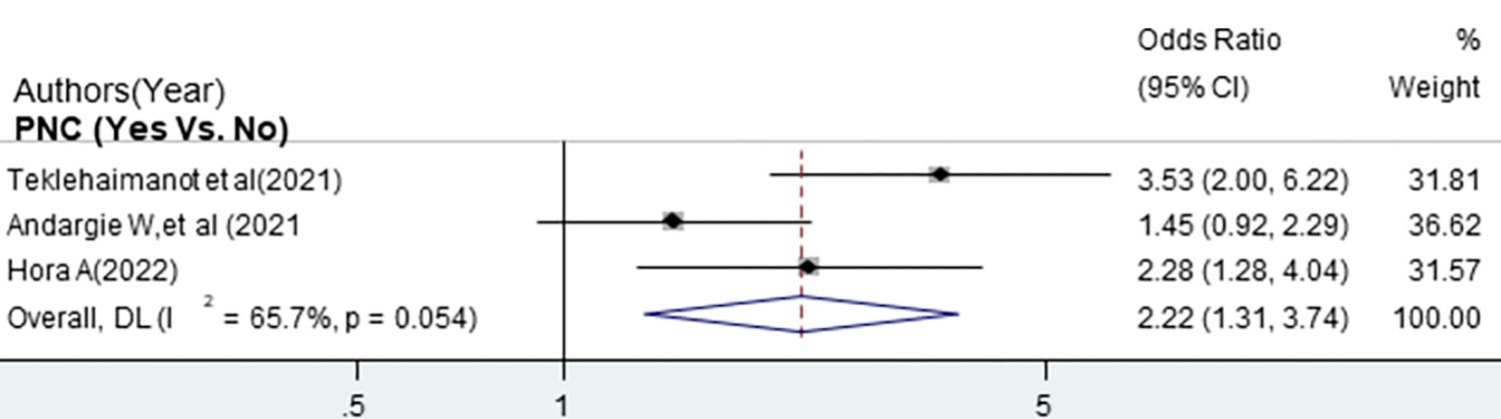
The pooled odds ratio of the association between PNC follow-up and good sunlight exposure practice among infant mothers pair in Ethiopia.

**Fig 6:**
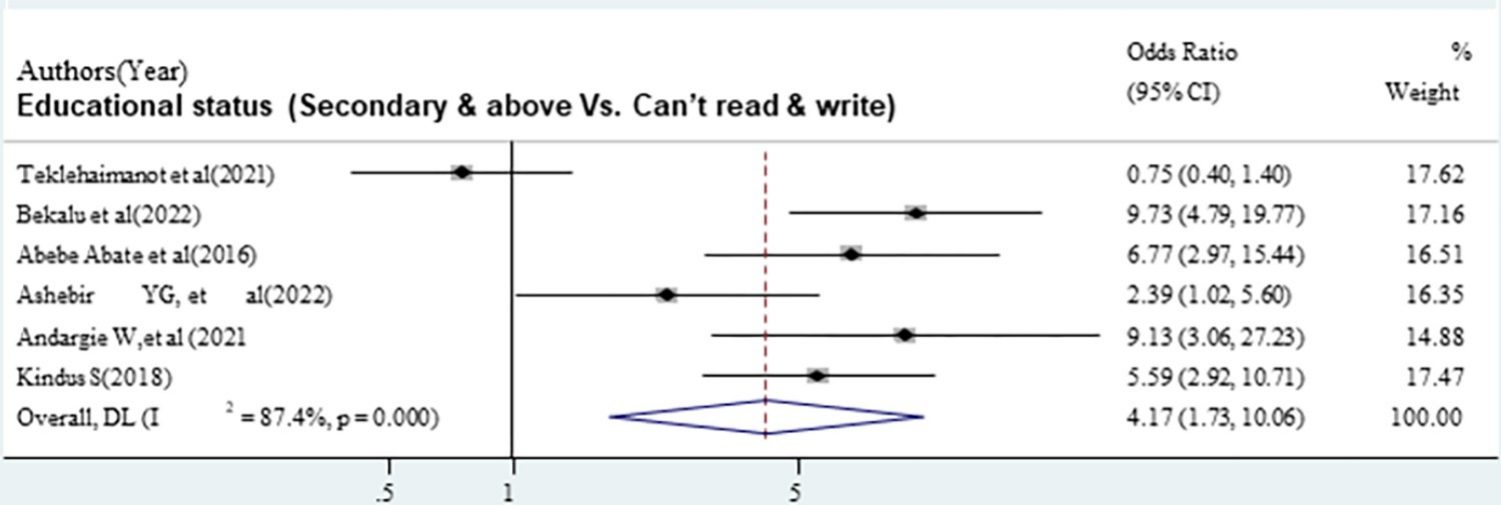
The pooled odds ratio of the association between mothers’ secondary and above educational status and good sunlight exposure practice in Ethiopia.

**Fig 7:**
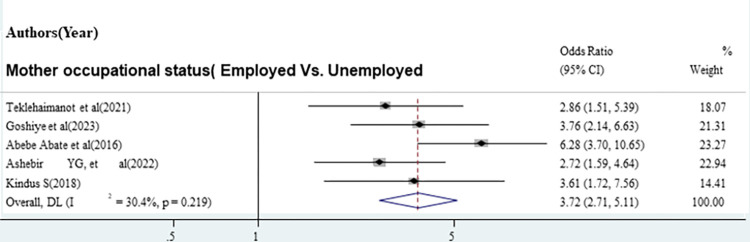
The pooled odds ratio of the association between employed mothers and good sunlight exposure practice in Ethiopia.

The odds of good sunlight exposure practice were 2.22 times higher among mothers who had PNC follow-up compared with mothers who had no PNC follow-up [OR = 2.22 (95% CI: 1.31, 3.47)]. Overall and within the study design, the value of I ^2^ was 65.7 (Fig 5). The odds of good sunlight exposure practice were 4.17 times higher among mothers with secondary and above educational status compared with mothers with can’t read and write [OR = 4.17, (95% CI: 1.73, 10.06)]. Overall and within the study design, the value of I ^2^ was high (Fig 6). The odds of good sunlight exposure practice were 3.72 times higher among employed mothers compared to those unemployed mothers [OR = 372, (95% CI: 2.71, 5.11)]. Overall and within the study design, the value of I ^2^ was low (Fig 7).

**Fig 8:**
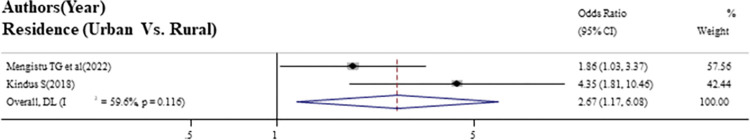
The pooled odds ratio of the association between the not fear of sunlight exposure and good sunlight exposure practice among infant-mother pairs in Ethiopia.

**Fig 9:**
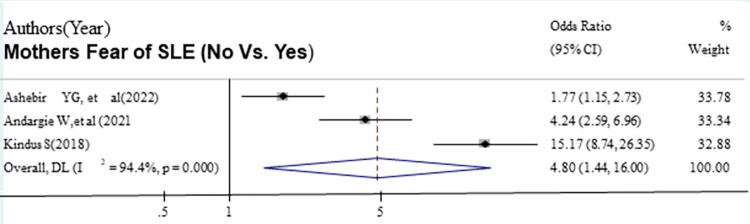
The pooled odds ratio of the association between urban residence and good sunlight exposure mong the infant mother pair in Ethiopia.

The odds of good sunlight exposure were 2.67 times higher among urban residence mother-infant pairs compared with mother-infant pairs who reside in rural areas [OR = 2.67, (95% CI: 1.17, 6.08)] (Fig 8). The odds of good sunlight exposure were 4.80 times higher among mothers with no fear of sunlight exposure compared with mothers with fear of sunlight exposure [OR = 4.08, (95% CI: 1.44, 16.00)] (Fig 9). However, we found that a woman who had ANC follow-up was not associated with good sunlight exposure practice compared to mothers who had no ANC follow-up.

## Discussion

Good sunlight exposure practice has a significant role for infants because, with sufficient exposure to ultraviolet B (UVB) from sunlight, a healthy person should be able to synthesize all of their vitamin D requirements in their skin. Therefore, the objective of this review was to estimate the pooled prevalence of good sunlight exposure practice and its associated factors among infants in Ethiopia.

According to this meta-analysis, the overall pooled prevalence of good sunlight exposure practices among infants in Ethiopia was 53.75% (95%CI: 46.84, 60.66). The finding of this study reveals that more than half of the infants were adequately exposed to sunlight in Ethiopia. This finding was consistent with other studies conducted in the UK (56%), and New Zealand (55.9%) [36, 37]. Vitamin D insufficiency continues to be a significant public health issue worldwide, regardless of the presence of sunlight [38]. Above 50% of healthy individuals were vitamin D sufficient, whereas not meeting the recommended dietary goal [39]. Exposure to sunlight is the most reasonable way to address the widespread issue of vitamin D deficiency. Furthermore, fortification or supplementation cannot fully substitute for sun exposure [40]. As per the Practical Cooking and Feeding Guide for Ethiopian Children 6–23 Months Old, it is recommended to have your baby spend 20 to 30 minutes in the sun each day to promote healthy bone growth and overall well-being by ensuring adequate vitamin D intake [41].

According to this study finding, the odds of good sunlight exposure practice were 2.22 times higher among mothers who had PNC follow-up compared with mothers who had no PNC follow-up. This might be because, during postnatal care follow-up, mothers receive guidance on the significance of sunlight exposure for their babies. It is essential to communicate the need for behaviour change to increase exposure to ultraviolet light in order to prevent vitamin D deficiency in countries with tropical climates, such as Ethiopia [42]. Mothers who lack adequate knowledge about new-born care and rely on traditional childcare methods may inadvertently harm or impair their new-borns [43].

The pooled odds of good sunlight exposure practice were 4.17 times higher among mothers with secondary and above educational status compared with mothers with couldn’t read and write. This could be attributed to the fact that in the Ethiopian context, mothers who have received education are more likely to comprehend and implement nutritional knowledge, as they are typically responsible for caring for infants and managing household activities, including ensuring exposure to sunlight. Educated mothers tend to be well-informed, have greater access to health-related information, and experience fewer challenges in understanding guidance from healthcare providers compared to those who are not educated [44].

The pooled odds of good sunlight exposure practice were 3.72 times higher among employed mothers compared to unemployed mothers. Most of the time, the employed mother is educated, being the main component that affects development and economic growth. Maternal education influences child health, which is an important factor in predicting well-being and productivity in adult life. Also, greater maternal education translates into greater healthcare utilization, including formal prenatal visits [45].

Urban residence mother-infant pairs had 2.67 times higher odds of good sunlight exposure compared to mother-infant pairs living in rural areas. This could be due to easier access to health facilities and nutrition information through various media in urban areas. Surprisingly, even rural agricultural labourers, who spend a significant amount of time outdoors with a large body surface area exposed, have reported a high prevalence of vitamin D deficiency ranging from 44–70% [46].

The pooled odds of good sunlight exposure were 4.80 times higher among mothers with no fear of sunlight exposure compared with mothers who fear sunlight exposure. This might be due to the mother not fearing the risk of sunlight exposure; the mother can adequately expose the child to sunlight. This is supported by the evidence when the mothers affected by cultural practices and beliefs to prevent evil eye not exposure to sunlight [47, 48].

### Strengths and limitations of the study

We try to use different search engines to address all data sets including unpublished studies to estimate the pooled prevalence of sunlight exposure practice and its determinants. Even though the analysis had strengths, it has certain limitations: The heterogeneity was not completely fixed in the final random effect model. These findings will have vital implications for program planners, policymakers, and healthcare providers to design nutrition intervention programs accordingly. Measurements for the level of practice were taken from each primary study with different operational definitions.

## Conclusion

The pooled prevalence of good sunlight exposure practice among infants is low. Being urban residents, PNC follow-up, mothers’ educational status and fear of sunlight exposure status were found to be associated with good sunlight exposure practices among infants. The governments and relevant stakeholders should develop and implement effective interventions to increase the prevalence of good sunlight exposure practices. Therefore health professionals create awareness for mothers about the importance of sunlight exposure and to increase follow postnatal care, especially for rural residence mothers.

## Supporting information

S1 Table PRISMA checklist. (DOCX)

S2 Table The datasets used/analysed in this systematic review and meta-analysis. (XLSX)
